# Molecular Imaging, Radiochemistry, and Environmental Pollutants

**DOI:** 10.2967/jnumed.122.265209

**Published:** 2023-08

**Authors:** Samantha Delaney, Joni Sebastiano, Brian M. Zeglis, Outi M. Keinänen

**Affiliations:** 1 Department of Chemistry, Hunter College, City University of New York, New York, New York;; 2 Department of Radiology, Memorial Sloan Kettering Cancer Center, New York, New York;; 3 Ph.D. Program in Biochemistry, Graduate Center of City University of New York, New York, New York;; 4 Ph.D. Program in Chemistry, Graduate Center of City University of New York, New York, New York;; 5 Department of Radiology, Weill Cornell Medical College, New York, New York; and; 6 Department of Chemistry, University of Helsinki, Helsinki, Finland

**Keywords:** environmental pollutants, molecular imaging, PET imaging, SPECT imaging, fluorescence imaging

## Abstract

The worldwide proliferation of persistent environmental pollutants is accelerating at an alarming rate. Not surprisingly, many of these pollutants pose a risk to human health. In this review, we examine recent literature in which molecular imaging and radiochemistry have been harnessed to study environmental pollutants. Specifically, these techniques offer unique ways to interrogate the pharmacokinetic profiles and bioaccumulation patterns of pollutants at environmentally relevant concentrations, thereby helping to determine their potential health risks.

Over the last half century, human activity has caused a devastating rise in environmental pollution. The scientific community has sounded the alarm about the potential effects of certain pollutants—including micro- and nanosized plastics, metal oxides, and per- and poly-fluoroalkyl substances (PFAS)—on human health, especially in light of recent studies showing these compounds to have become ubiquitous in human biological systems (1–5). For example, micro- and nanosized plastics have recently been isolated from human blood, fecal matter, and placental tissue. As science seeks to unravel the toxicologic and biological effects of pollutants, understanding their pharmacokinetic profiles and bioaccumulation patterns is critical. To this end, scientists have begun to turn to molecular imaging, as it offers an accurate and noninvasive way to monitor the in vivo behavior of pollutants in living systems and, in so doing, identify potential sites of interaction and toxicity.

In this review, we discuss recent efforts to use molecular imaging and radiochemistry to study environmental pollutants. We are not the first to cover this topic; several excellent reviews have been published on work at the intersection of molecular imaging, radiochemistry, and environmental science (6–9). Although the field remains young, it has grown in recent years and—in our estimation—is poised to break out into the mainstream soon. We will cover how various molecular imaging and radiochemical methods have been used to study the pharmacokinetic profiles of five types of environmental pollutants: micro- and nanoplastics, PFAS, metal oxides, particulate matter (PM), and graphene. Ultimately, we hope that this review will shed light on how these techniques offer unique tools for probing the pharmacological behavior and biological impact of environmental pollutants and thus may help determine how to mitigate their risks to human health.

## Micro- and Nanoplastics

**FIGURE 1.**
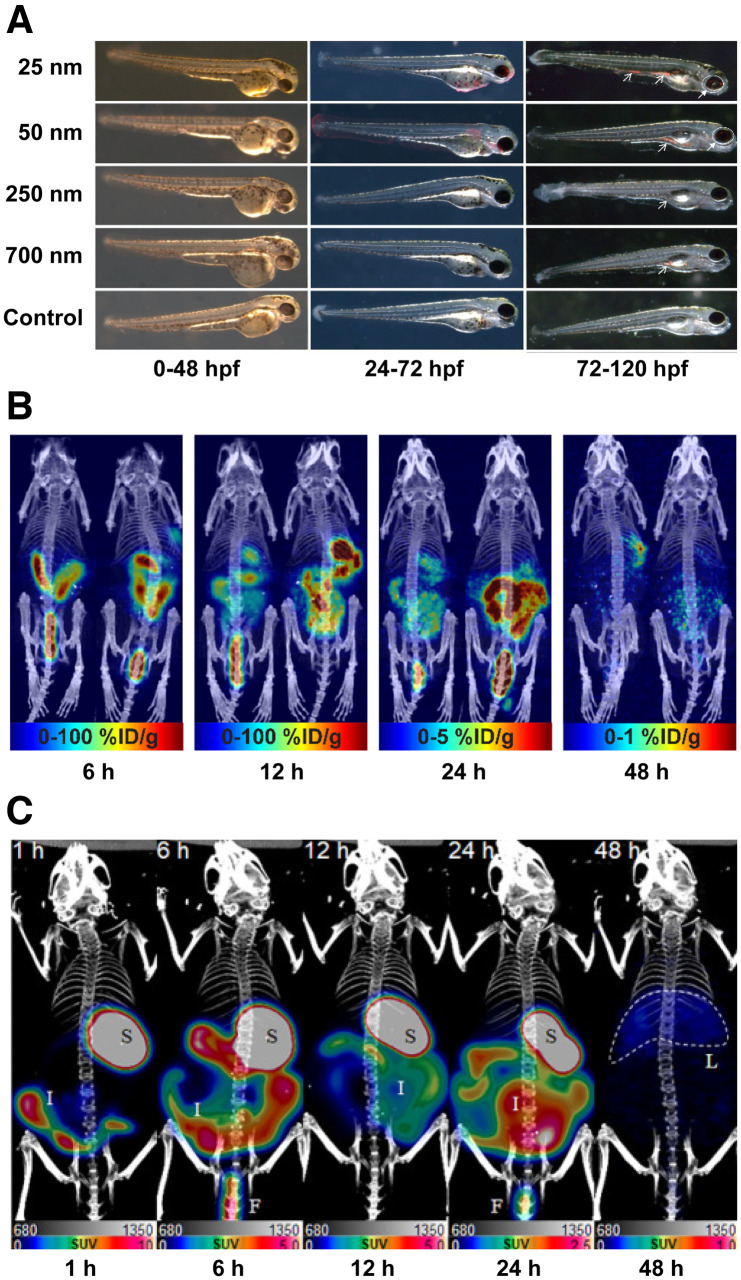
(A) Fluorescence microscopy images of different-sized polystyrene particles (25, 50, 250, and 700 nm) in zebrafish embryos (13). (B) Maximum-intensity-projection PET/CT images of mice at 6, 12, 24, and 48 h after administration of [^89^Zr]Zr-desferrioxamine-polystyrene (20 nm; 1.85 MBq; 0.1 mg) via oral gavage (16). (C) Maximum-intensity-projection PET/CT images of mice at 1, 6, 12, 24, and 48 h after oral administration of [^64^Cu]Cu-DOTA-polystyrene (200 nm; 4.81 MBq; 57.8 μg) (17). %ID = percent injected dose; hpf = hours postfertilization.

Contamination of the environment with microplastics (<5 mm in diameter) and nanoplastics (<100 nm in diameter) has grown substantially over the past few decades, and numerous studies have linked plastic pollution with adverse health effects in humans. Indeed, high levels of micro- and nanoplastic exposure have been associated with inflammatory effects as well as respiratory and cardiovascular diseases (1*,*^2^). However, the in vivo fate of micro- and nanoplastics in mammalian systems is relatively poorly understood, opening the door for molecular imaging to be used in this arena. In 2017, Deng et al. administered fluorophore-bearing polystyrene microplastics (5 and 20 μm) to mice via oral gavage (10). Although these particles could not be tracked in vivo because of the low tissue penetration of the fluorescent signal, ex vivo methods revealed accumulation of the particles in the liver, kidneys, and gut. In addition, the cohort that received the highest dose of microplastic particles exhibited inflamed livers, decreased body weights, decreased adenosine triphosphate levels, and increased lactate dehydrogenase activity. In another study, the translocation and fetal deposition of 20 nm rhodamine-labeled polystyrene nanoparticles were interrogated in pregnant rats (11). Again, the poor tissue penetration of visible light fluorescence prevented in vivo imaging, but ex vivo detection methods identified plastics in the maternal lung, heart, and spleen as well as several fetal tissues, suggesting translocation of the plastics through the placenta. Fluorophore-labeled polystyrene particles have, however, been harnessed for in vivo tracking in another, more transparent animal model: zebrafish. Pitt et al. reported that fluorescent polystyrene nanoparticles (50 nm) accumulated in the chorion of developing zebrafish embryos, resulting in toxicity due to the particles’ penetration of the nutrient-rich yolk sac (12). These results align with those of van Pomeren et al., who studied the distribution of fluorophore-bearing polystyrene nanoparticles of several sizes (25, 50, 250, and 700 nm) in zebrafish embryos at various stages of development (Fig. 1A) (13).

Several methods for the radiolabeling of micro- and nanoplastics have been developed to facilitate their study via autoradiography and nuclear imaging. For example, Al-Sid-Cheikh et al. used whole-body autoradiography to determine that ^14^C-labeled plastics accumulated in the muscles, gonads, mantle, gills, intestines, and kidneys of scallops, with the highest concentration detected in the hepatopancreas (14*,*^15^). Recently, our group reported the first (to our knowledge) study in which PET was used to track radiolabeled micro- and nanoplastic particles (16). In this work, ^89^Zr was used to radiolabel micro- and nanosized polystyrene particles (20 nm, 220 nm, 1 μm, and 6 μm) modified with the chelator desferrioxamine. PET imaging experiments revealed that after administration via oral gavage, these ^89^Zr-labeled radioplastics traveled through the gastrointestinal tract without reaching the systemic circulation (Fig. 1B). More recently, Im et al. labeled 200 nm polystyrene microplastic particles with the positron-emitting radiometal ^64^Cu and used PET to follow their in vivo behavior in mice (Fig. 1C) (17). In this case, the data revealed high activity concentrations in the stomach, intestines, and liver. However, this phenomenon could be due to translocation of the radioplastics or inadvertent release and redistribution of [^64^Cu]Cu^2+^, because the chelator used in this system—DOTA—has been shown to be an inadequate platform for the stable in vivo sequestration of Cu^2+^ (18). Finally, radioiodination methods have recently been developed for polyvinylchloride particles (19). Although this technology has not yet been used to facilitate nuclear imaging, it is nonetheless exciting, as it could pave the way for PET imaging studies with the positron-emitting isotope of iodine, ^124^I.

## PFAS

PFAS are classes of organofluorine compounds widely used in both industrial and consumer applications. From a chemical standpoint, PFAS offer numerous advantages—such as high inertness due to the strength of their carbon-fluorine bonds—but they come with a significant drawback: unfavorably long half-lives within environmental and biological systems (4). Recent monitoring studies have confirmed the pervasive presence of PFAS in water and land environments, as well as in aquatic species at all levels of the food web. Human exposure to PFAS has been linked to several negative health effects, such as impaired immunological function, chronic autoimmune diseases, hepatotoxicity, cancer, decreased fertility, and toxic developmental effects (20*,*^21^). Because of their small size, PFAS cannot reliably be labeled with fluorophores without disturbing their in vivo behavior. Instead, the field has turned to radiochemistry. Bogdanska et al., for example, administered ^35^S-labeled perfluorooctane sulfonate orally to adult mice and used whole-body autoradiography and scintillation counting to study the compound’s biodistribution, ultimately finding the highest concentrations in the liver, bone, blood, skin, and muscle (22). More recently, Bartels et al. labeled perfluorooctanoic acid (PFOA), perfluorohexanoic acid, and perfluorobutanoic acid with the positron-emitting radiohalogen ^18^F (23). Ex vivo biodistribution data collected 4 h after the intravenous administration of these radiopollutants revealed that the highest concentrations of [^18^F]F-PFOA and [^18^F]F-perfluorohexanoic acid were in the liver, whereas that of [^18^F]F-perfluorobutanoic acid was in the stomach. As an aside, it is worth noting that all of the mouse tissues examined in the aforementioned studies contained background levels of unlabeled PFAS, a result that aligns with postmortem analyses of human tissues.

**FIGURE 2.**
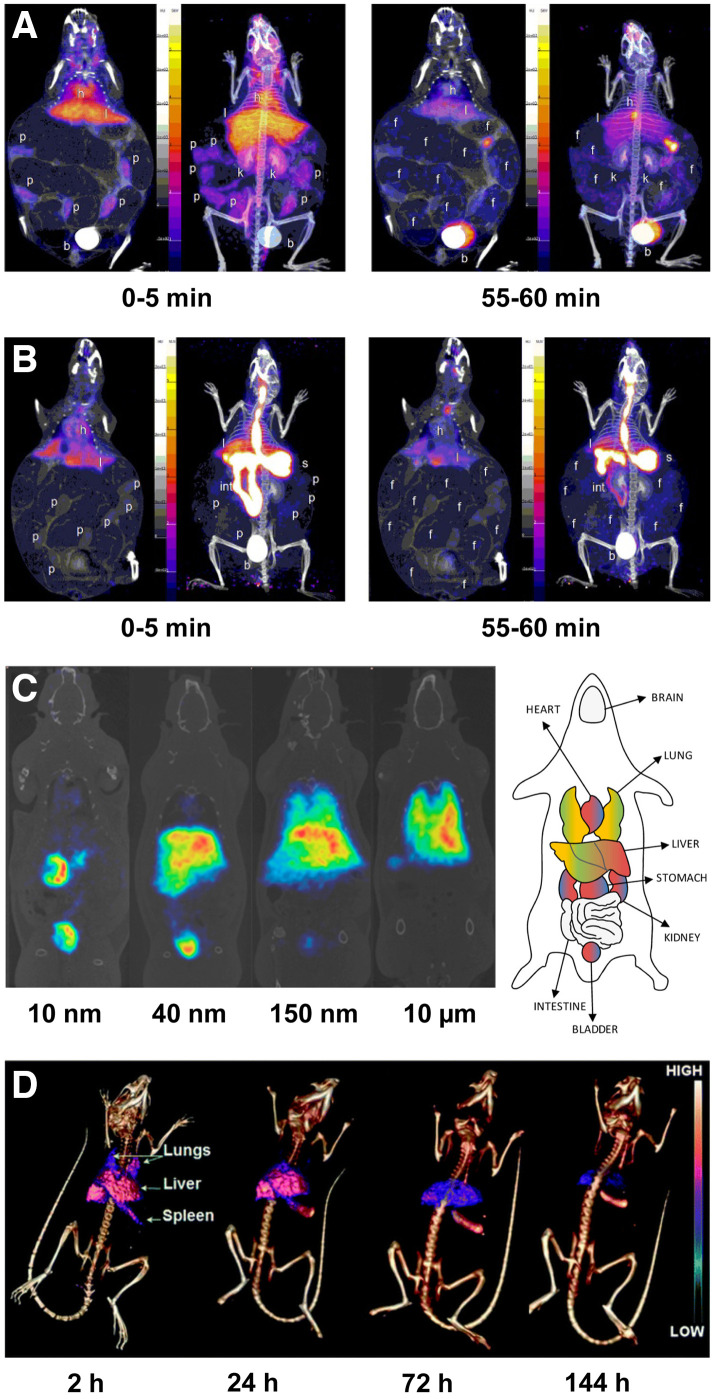
(A and B) Representative PET images illustrating uptake of [^18^F]PFOA in mice at 2 time points after administration via tail vein injection (4.44 MBq) (A) and oral gavage (3.7 MBq) (B). b = bladder; f = fetus; h = heart; int = intestine; k = kidney; l = liver; p = placenta; s = stomach. (Reprinted with permission of (25).) (C) PET images of mice at 60 min after intravenous administration of 10 nm, 40 nm, 150 nm, or 10 μm of ^13^N-labeled Al_2_O_3_ nanoparticles (10–15 MBq, 5–8 mg). (Reprinted with permission of (27).) (D) SPECT/CT images of mice at 2, 24, 72, and 144 h after intravenous administration of ^141^Ce-labeled cerium oxide nanoparticles (6.7 MBq, 3.6 nmol). (Reprinted with permission of (29).)

Since high levels of exposure to PFAS can lead to birth defects, researchers have been particularly interested in investigating the fate of PFAS in gravid mice and in their fetuses and pups. In a study by Borg et al., gravid mice were administered ^35^S-labeled perfluorooctane sulfonate either intravenously or orally, and the bioaccumulation of the radiopollutant was subsequently measured in the dams, fetuses (on gestational days 18 and 20), and pups (on postnatal day 1) via whole-body autoradiography and scintillation counting (24). Surprisingly, the activity concentrations of [^35^S]S-perfluorooctane sulfonate in the blood of the fetuses and pups were 1.1–2.3 times higher than that in the dam’s blood. The activity concentrations in the livers of the fetuses were significantly lower than that in the maternal liver but were still about 2.5-fold higher than that in the maternal blood. Autoradiography also revealed heterogeneous uptake of [^35^S]S-perfluorooctane sulfonate in the brains of fetuses and pups. In another study, Bartels et al. used dynamic PET and biodistribution studies to explore the biologic fate of two ^18^F-labeled PFAS—[^18^F]F-PFOA and [^18^F]F-perfluorohexanoic acid—in pregnant mice after intravenous and oral administration (25). PET images revealed uptake of both compounds in the placentae, though the rate of accumulation was slower after oral administration (Figs. 2A and 2B). The radiolabeled PFAS were found in all tissues examined, with the highest concentrations in the blood (after intravenous injection) and in the gastrointestinal tract and lungs (after oral administration). Taken together, the studies described in this section effectively illustrate the intrinsic advantages and disadvantages of certain radionuclides for pollutant studies. Although the 87.4-d half-life of ^35^S allows for longitudinal monitoring, its emissions do not permit in vivo imaging; ^18^F (half-life, 109.8 min), on the other hand, can be imaged via PET, but its short half-life limits follow-up times.

## Metal Oxides

Nanoparticulate metal oxides have been used in a vast array of applications, including food additives, cosmetics, semiconductors, electronics, and medicine. This widespread use has inadvertently led to their proliferation as environmental pollutants, which, in turn, has fueled the study of their potential long-term biological effects. Radionuclides are the most common reporter for the in vivo study of metal oxides. Along these lines, Pérez-Campaña et al. have developed a robust proton beam methodology for the production of metal oxides radiolabeled with short-lived positron emitters (26–28). For example, they used the direct irradiation of ^18^O-enriched Al_2_O_3_ to generate ^18^F-labeled particles that were then administered intravenously to rats (26). PET imaging revealed that most of the aluminum oxide nanoparticles accumulated in the liver, though considerable uptake was also observed in the lungs, heart, kidneys, urine, and stomach. In a similar study, the same group produced ^18^F-labeled TiO_2_ nanoparticles and administered them to rats both orally and intravenously (28). Subsequent PET imaging revealed the accumulation of these nanoparticles in the upper gastrointestinal tract and liver on oral and intravenous administration, respectively. Finally, Pérez-Campaña et al. have also investigated the effect of particle size on the biodistribution of metal oxides. After the production of ^13^N-labeled Al_2_O_3_ nanoparticles of different sizes via proton beam activation, in vivo biodistribution results showed that larger nanoparticles (i.e., 40 nm, 150 nm, and 10 μm) accumulated in the liver and lungs whereas smaller nanoparticles (i.e., 10 nm) accreted in excretory organs such as the bladder and kidneys (Fig. 2C) (27).

Shifting gears, a study performed by Yang et al. leveraged longer-lived radionuclides and SPECT to investigate the in vivo behavior of cerium oxide nanoparticles in mice up to 1 wk after intravenous injection (29). To this end, cerium oxide nanoparticles were radiolabeled with ^141^Ce, ^111^In, or ^65^Zn. SPECT imaging revealed high levels of accumulation in the lungs, liver, and spleen at early time points after intravenous administration (Fig. 2D). The particles gradually cleared from the lungs over time but remained in the liver and spleen until the end of the observation period.

## PM

**FIGURE 3.**
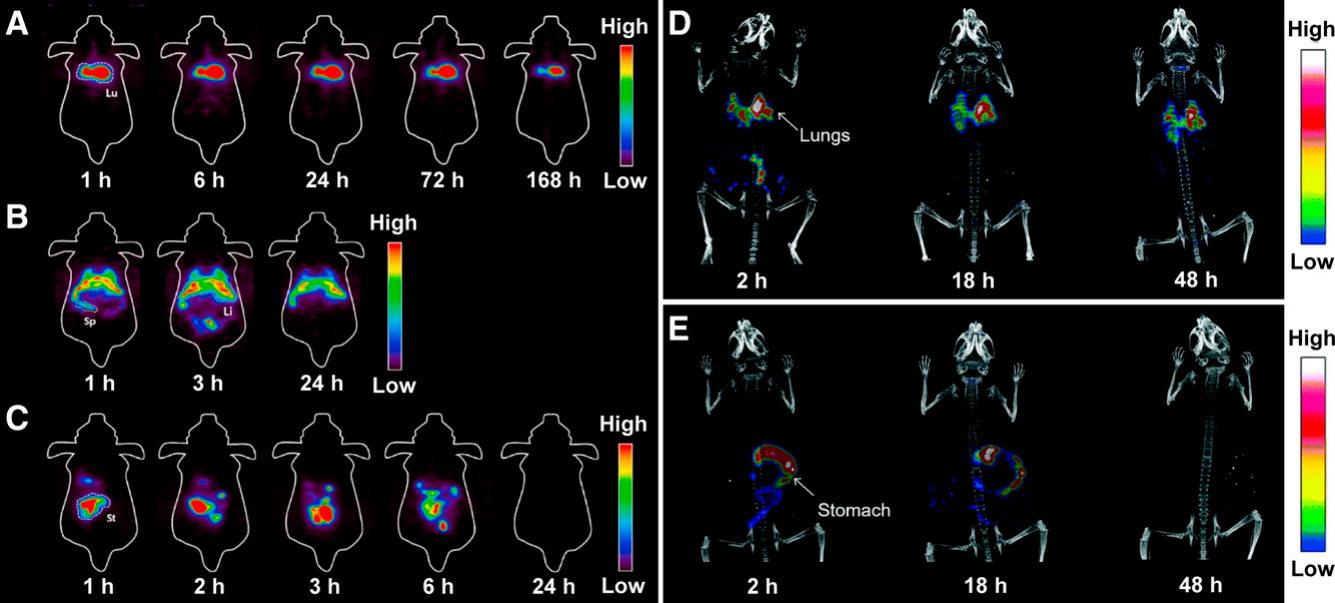
(A–C) Representative PET images of mice at 168 h after intratracheal administration (A), 24 h after oral administration (B), and 24 h after intravenous injection (C) of [^89^Zr]Zr-desferrioxamine-pyr-diesel PM (1.85 MBq). Li = liver; Lu = lungs; Sp = spleen; St = stomach. (Reprinted with permission of (3).) (D–E) Whole-body SPECT/CT images of mice at 2, 18, and 48 h after intratracheal (D) and oral (E) administration of ^125^I-labeled diesel exhaust particulates (3.7 MBq) (30).

*Particulate matter* is a general term for airborne particulate pollutants that are small enough to cause serious health issues on inhalation. PM, a mixture of solids and liquids that can contain hundreds of different chemicals, is categorized into 2 groups based on the size of the particles: those with a diameter of less than 10 μm (PM_10_) and those with a diameter of less than 2.5 μm (PM_2.5_). Recent years have played witness to a surge of reports linking PM to chronic respiratory issues, cardiovascular disease, cancer development, and even—in severe cases—death. In an effort to study this, Park et al. used ^89^Zr to radiolabel an analog of pyrene that was then used to prepare a suspension of diesel PM, a subset of PM that is emitted by diesel engines (3). This radioactive diesel PM (average size, ∼200 nm) was then administered to mice orally, intratracheally, or intravenously. PET imaging revealed that the mice that received the ^89^Zr-labeled diesel PM orally excreted the particles within a day (Fig. 3A). In contrast, the particles administered via the other 2 routes were retained in several organs. More specifically, intratracheally administered particles accumulated principally in the lungs (Fig. 3B), whereas those administered intravenously accreted in the heart, liver, and spleen (Fig. 3C). A few years earlier, Lee et al. leveraged SPECT imaging to track diesel exhaust particulates in vivo (30). In this investigation, an ^125^I-labeled variant of pyrene was incorporated in diesel PM (average size, ∼200 nm) and administered intratracheally to mice. SPECT imaging and biodistribution experiments revealed that most of the radiolabeled particulates persisted in the lungs (Fig. 3D), reinforcing the dangers of pulmonary exposure in humans. Recently, Li et al. developed a fluorescence imaging method to determine the rate and pattern of PM_2.5_ deposition in murine lung tissue (31). Two sizes of fluorescent latex microspheres—0.2 and 2 μm—were used to simulate the small and large components of PM_2.5_ in the air. The deposition patterns were nonuniform, revealing higher rates of deposition in the acinar area than those predicted using the current state-of-the-art deposition model. Pan et al. embarked on the study of another particulate material when they modified SiO_2_ with ethyl cellulose (32). This modification provided a facile route to radioiodination, and ^131^I-labeled ethyl cellulose/SiO_2_ (1.2 μm) was synthesized and administered to rats via inhalation. SPECT images at 6 and 24 h after inhalation showed high initial uptake in the lungs that decreased significantly with time.

## Graphene

**FIGURE 4.**
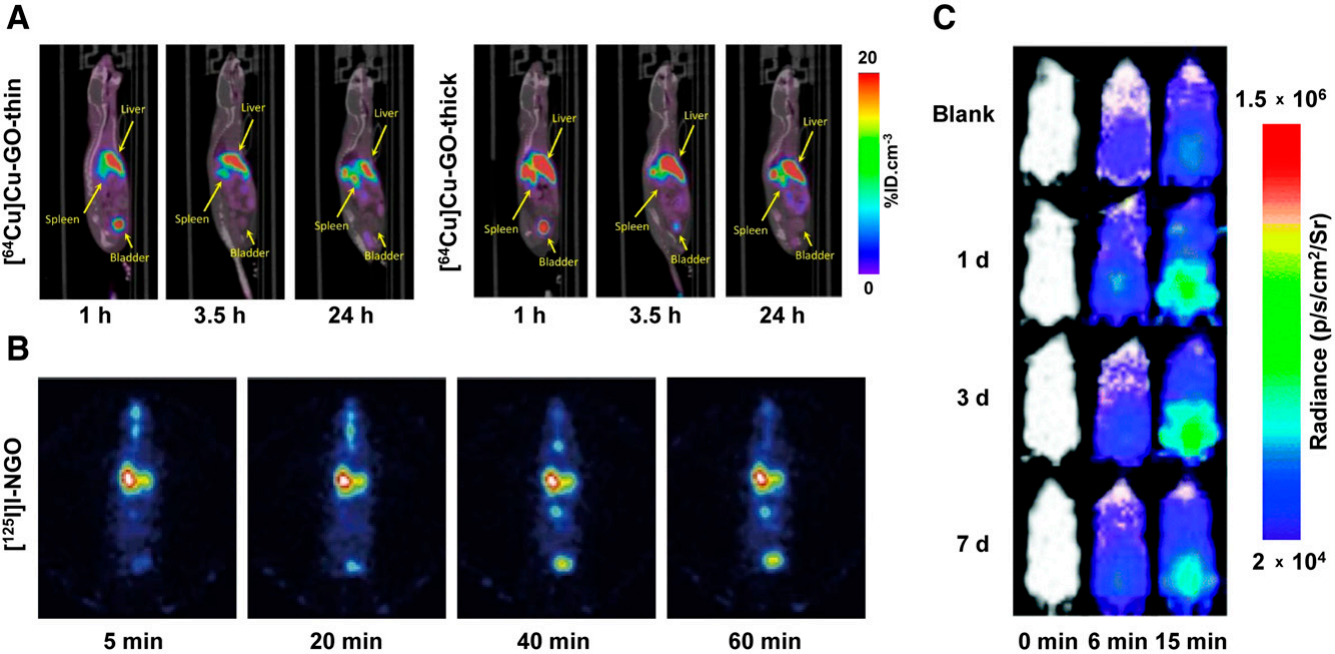
(A) Whole-body PET/CT images of mice at 1, 3.5, and 24 h after intravenous administration of 2.51–4.55 MBq (50 μg) of [^64^Cu]Cu-GO-thin (left) or [^64^Cu]Cu-GO-thick (right). (Reprinted with permission of (34).) (B) SPECT images of mice at 5, 20, 40, and 60 min after intratracheal administration of [^125^I]I-nanoscale GO (1.85 MBq) (37). (C) Bioluminescence images of mice at 1, 3, and 7 d after intravenous administration of Hg^2+^ (0.02 μmol) and facilitated using intraperitoneally injected Hg^2+^-sensitive probe. (Reprinted with permission of (38).) %ID = percent injected dose.

Graphene and its derivatives are relative newcomers in biological research. However, their attractive physicochemical properties and high biocompatibility have fueled a rise in their use across several industries. Graphene nanomaterials are 2-dimensional lattices of carbon atoms that, once released into the environment, are prone to aggregation and settlement into sediment layers (33*,*^34^). It is estimated that the world’s production of graphene will reach 3,800 tons by 2027, so gaining an improved understanding of the pollutant’s in vivo behavior is of paramount importance. In one study, Jasim et al. functionalized graphene oxide (GO) sheets with DOTA, radiolabeled them with ^111^In, and found that the highest levels of accumulation after intravenous injection were in the urine and spleen (35). In a follow-up study, the same team investigated the effect of the thickness of graphene on its biodistribution by labeling both thick and thin DOTA-bearing GO sheets with ^64^Cu (∼0.8 μm in the largest dimension; thin flakes = 4–8 nm thick; thick flakes = 20–50 nm thick) (34). The intravenous injection of both variants produced rapid urinary excretion as well as high levels of persistent uptake in the liver and spleen, though the thick sheets accumulated in these tissues more quickly (Fig. 4A). Jasim et al. also investigated the biodistribution profiles of ^111^In-labeled GO sheets with 3 different sizes in lateral dimension: 1–35 μm, 30–1,900 nm, and 10–550 nm (36). All 3 varieties accumulated in the liver and spleen and were excreted through the urine. Interestingly, however, the largest sheets exhibited lung uptake that was not seen with the smaller variants.

Shifting to other laboratories, Li et al. radiolabeled nanoscale GO (10–800 nm) with ^125^I and administered it intratracheally to mice (37). SPECT imaging and biodistribution experiments revealed high concentrations of [^125^I]I-nanoscale GO in the lungs at early time points, followed by a gradual decrease over several hours (Fig. 4B). Later, Mao et al. administered ^14^C-labeled few-layer graphene (60–590 nm in the largest dimension; 1–4 nm thick) to mice intratracheally and used ex vivo measurements to track its biodistribution over 28 d (38). Although the ^14^C-labeled few-layer graphene was increasingly excreted via the feces, almost 50% of the dose remained in the lungs after 4 wk. Lu et al. also synthesized ^14^C-labeled few-layer graphene but added their construct to water and sediment and subsequently quantified its bioaccumulation and biodistribution in loaches (*M. anguillicaudatus*), a type of freshwater, bottom-dwelling fish that ingests large amounts of sediment and are consumed by humans and other water-dwelling animals (33). After only 72 h in a container with sediment mixed with ^14^C-labeled few-layer graphene, radioactivity was found in the gut and liver of the loaches. In a very recent proof-of-principle study, autoradiography and mass spectrometry imaging were used to visualize ^14^C-labeled GO particles (50–400 nm, 1 nm thick) in mouse tissue sections 15 min after intravenous injection (39). The study validated mass spectrometry imaging as an efficient tool for the determination of the microscopic distribution of GOs. The four organs collected—lungs, liver, spleen, and kidneys—contained more than 96% of the total injected dose, with the highest uptake in the liver (>50% injected dose).

## Other Pollutants

Although much of the research at the intersection of molecular imaging, radiochemistry, and environmental pollutants has focused on the five types of contaminants discussed above, the literature contains a handful of reports in which these techniques have been used to study other pollutants. Al-Sid-Cheikh et al., for example, created ^110m^Ag-labeled silver nanoparticles and studied their bioaccumulation in Arctic char via quantitative whole-body autoradiography (40). ^110m^Ag-labeled silver nanoparticles are widely used in the production of nanomaterials as well as consumer products (41). In this study, uptake was observed in several organs on exposure of the fish via three administration methods—waterborne exposure, intravenous injection, and force-feeding—with the highest overall accumulation seen after intravenous exposure. Waterborne exposure, the most environmentally relevant method, yielded accretion levels lower than those produced by the other methods.

Using a dramatically different approach, Ke et al. created a bioluminescence imaging probe for tracking the accumulation of mercury in mice (42). Mercury is a naturally occurring environmental pollutant that can cause DNA damage and a wide variety of systemic diseases in humans and animals. The team synthesized a probe that reacts with Hg^2+^ to release luciferin, a substrate for luciferase, resulting in bioluminescence. This Hg^2+^-activated probe was administered intraperitoneally to transgenic FVB-*luc+* mice—which ubiquitously express firefly luciferase—1, 3, and 7 d after the intravenous injection of Hg^2+^. The noninvasive visualization of the bioluminescence signal provided insight into the accumulation and clearance of Hg^2+^: by 7 d after administration, the Hg^2+^-driven luminescent signal significantly decreased (Fig. 4C).

## CONCLUSION

Despite the efforts of many, environmental pollution is a persistent, stubborn, and—in many cases—worsening problem. Many pollutants pose threats to human health, and thus it is critical to characterize the potential toxicity of these environmental contaminants. An important step in this process is determining where these pollutants go in the body after exposure via different routes. Molecular imaging and radiochemistry can be indispensable in this regard, as they can help determine the pharmacokinetic profiles of pollutants at environmentally relevant doses. As methods for the labeling and tracking of environmental pollutants are disseminated, we expect to see a surge in studies that use molecular imaging to examine these potentially hazardous compounds. Among the methods discussed in this work, we believe that nuclear imaging has the greatest potential. Indeed, the advantages of nuclear imaging include high sensitivity that allows for the interrogation of pollutants at environmentally relevant concentrations, nearly unlimited tissue penetration, the opportunity for long-term longitudinal studies, and a wide array of robust labeling methods using commercially available radionuclides. It is our hope that the pioneering work that we have described here helps usher in a new era in which molecular imaging and radiochemistry become standard tools for the in vivo study of environmental pollutants.

## DISCLOSURE

Financial support was received from the NIH (F31CA275334 to Samantha Delaney; K99ES034053 to Outi Keinänen; and R01CA240963, R01CA204167, and R01CA244327 to Brian Zeglis), the Academy of Finland (331659 to Outi Keinänen), and the Tow Foundation (Joni Sebastiano). No other potential conflict of interest relevant to this article was reported.

## References

[1] Stapleton PA. Microplastic and nanoplastic transfer, accumulation, and toxicity in humans. Curr Opin Toxicol. 2021;28:62–69. 34901583 10.1016/j.cotox.2021.10.001 PMC8654083

[2] López de las Hazas MC Boughanem H Dávalos A. Untoward effects of micro- and nanoplastics: an expert review of their biological impact and epigenetic effects. Adv Nutr. 2021;13:1310–1323. 10.1093/advances/nmab154 PMC9340974 34928307

[3] Park JE Lee JY Chae J. In vivo tracking of toxic diesel particulate matter in mice using radiolabeling and nuclear imaging. Chemosphere. 2023;313:137395. 36574577 10.1016/j.chemosphere.2022.137395

[4] Manojkumar Y Pilli S Rao PV Tyagi RD. Sources, occurrence and toxic effects of emerging per- and polyfluoroalkyl substances (PFAS). Neurotoxicol Teratol. 2023;97:107174. 36907230 10.1016/j.ntt.2023.107174

[5] Pietroiusti A Stockmann-Juvala H Lucaroni F Savolainen K. Nanomaterial exposure, toxicity, and impact on human health. Wiley Interdiscip Rev Nanomed Nanobiotechnol. 2018;10:e1513. 29473695 10.1002/wnan.1513

[6] Cresswell T Metian M Golding LA Wood MD. Aquatic live animal radiotracing studies for ecotoxicological applications: addressing fundamental methodological deficiencies. J Environ Radioact. 2017;178–179:453–460. 10.1016/j.jenvrad.2017.05.017 28629682

[7] Lanctôt CM Al-Sid-Cheikh M Catarino AI. Application of nuclear techniques to environmental plastics research. J Environ Radioact. 2018;192:368–375. 30045000 10.1016/j.jenvrad.2018.07.019

[8] Lee JY Mushtaq S Park JE Shin HS Lee SY Jeon J. Radioanalytical techniques to quantitatively assess the biological uptake and in vivo behavior of hazardous substances. Molecules. 2020;25:3985. 32882977 10.3390/molecules25173985 PMC7504758

[9] Fan Y Pan D Yang M Wang X. Radiolabelling and in vivo radionuclide imaging tracking of emerging pollutants in environmental toxicology: a review. Sci Total Environ. 2023;866:161412. 36621508 10.1016/j.scitotenv.2023.161412

[10] Deng Y Zhang Y Lemos B Ren H. Tissue accumulation of microplastics in mice and biomarker responses suggest widespread health risks of exposure. Sci Rep. 2017;7:46687. 28436478 10.1038/srep46687 PMC5402289

[11] Fournier SB D’Errico JN Adler DS. Nanopolystyrene translocation and fetal deposition after acute lung exposure during late-stage pregnancy. Part Fibre Toxicol. 2020;17:55. 33099312 10.1186/s12989-020-00385-9 PMC7585297

[12] Pitt JA Kozal JS Jayasundara N. Uptake, tissue distribution, and toxicity of polystyrene nanoparticles in developing zebrafish (*Danio rerio*). Aquat Toxicol. 2018;194:185–194. 29197232 10.1016/j.aquatox.2017.11.017 PMC6959514

[13] van Pomeren M Brun NR Peijnenburg WJGM Vijver MG. Exploring uptake and biodistribution of polystyrene (nano)particles in zebrafish embryos at different developmental stages. Aquat Toxicol. 2017;190:40–45. 28686897 10.1016/j.aquatox.2017.06.017

[14] Al-Sid-Cheikh M Rowland SJ Kaegi R Henry TB Cormier M-A Thompson RC. Synthesis of ^14^C-labelled polystyrene nanoplastics for environmental studies. Commun Mater. 2020;1:97.

[15] Al-Sid-Cheikh M Rowland SJ Stevenson K Rouleau C Henry TB Thompson RC. Uptake, whole-body distribution, and depuration of nanoplastics by the scallop *Pecten maximus* at environmentally realistic concentrations. Environ Sci Technol. 2018;52:14480–14486. 30457844 10.1021/acs.est.8b05266

[16] Keinänen O Dayts EJ Rodriguez C. Harnessing PET to track micro- and nanoplastics in vivo. Sci Rep. 2021;11:11463. 34075133 10.1038/s41598-021-90929-6 PMC8169765

[17] Im C Kim H Zaheer J. PET tracing of biodistribution for orally administered ^64^Cu-labeled polystyrene in mice. J Nucl Med. 2022;63:461–467. 34215675 10.2967/jnumed.120.256982 PMC8978192

[18] Boswell CA Sun X Niu W. Comparative in vivo stability of copper-64-labeled cross-bridged and conventional tetraazamacrocyclic complexes. J Med Chem. 2004;47:1465–1474. 14998334 10.1021/jm030383m

[19] Munir M Sholikhah UN Lestari E. Iodine-131 radiolabeled polyvinylchloride: a potential radiotracer for micro and nanoplastics bioaccumulation and biodistribution study in organisms. Mar Pollut Bull. 2023;188:114627. 36701974 10.1016/j.marpolbul.2023.114627

[20] Ehsan MN Riza M Pervez MN Khyum MMO Liang Y Naddeo V. Environmental and health impacts of PFAS: sources, distribution and sustainable management in North Carolina (USA). Sci Total Environ. 2023;878:163123. 37001657 10.1016/j.scitotenv.2023.163123

[21] Uhl M Schoeters G Govarts E. PFASs: what can we learn from the European Human Biomonitoring Initiative HBM4EU. Int J Hyg Environ Health. 2023;250:114168. 37068413 10.1016/j.ijheh.2023.114168

[22] Bogdanska J Borg D Sundström M. Tissue distribution of ^35^S-labelled perfluorooctane sulfonate in adult mice after oral exposure to a low environmentally relevant dose or a high experimental dose. Toxicology. 2011;284:54–62. 21459123 10.1016/j.tox.2011.03.014

[23] Bartels JL Aweda TA Rosenberg AJ Lunderberg DM Peaslee GF Lapi SE. Radiosynthesis and biological distribution of ^18^F-labeled perfluorinated alkyl substances. Environ Sci Technol Lett. 2017;4:211–215.

[24] Borg D Bogdanska J Sundström M. Tissue distribution of ^35^S-labelled perfluorooctane sulfonate (PFOS) in C57Bl/6 mice following late gestational exposure. Reprod Toxicol. 2010;30:558–565. 20656018 10.1016/j.reprotox.2010.07.004

[25] Bartels JL Fernandez SR Aweda TA. Comparative uptake and biological distribution of [^18^F]-labeled C6 and C8 perfluorinated alkyl substances in pregnant mice via different routes of administration. Environ Sci Technol Lett. 2020;7:665–671.

[26] Pérez-Campaña C Gómez-Vallejo V Martin A. Tracing nanoparticles in vivo: a new general synthesis of positron emitting metal oxide nanoparticles by proton beam activation. Analyst. 2012;137:4902–4906. 22957337 10.1039/c2an35863h

[27] Pérez-Campaña C Gómez-Vallejo V Puigivila M. Biodistribution of different sized nanoparticles assessed by positron emission tomography: a general strategy for direct activation of metal oxide particles. ACS Nano. 2013;7:3498–3505. 23473535 10.1021/nn400450p

[28] Pérez-Campaña C Sansaloni F Gómez-Vallejo V. Production of ^18^F-labeled titanium dioxide nanoparticles by proton irradiation for biodistribution and biological fate studies in rats. Part Part Syst Charact. 2014;31:134–142.

[29] Yang L Sundaresan G Sun M. Intrinsically radiolabeled multifunctional cerium oxide nanoparticles for in vivo studies. J Mater Chem B. 2013;1:1421–1431. 32260782 10.1039/c2tb00404f

[30] Lee CH Shim HE Song L. Efficient and stable radiolabeling of polycyclic aromatic hydrocarbon assemblies: in vivo imaging of diesel exhaust particulates in mice. Chem Commun (Camb). 2019;55:447–450. 30474665 10.1039/c8cc08304e

[31] Li D Li Y Li G Zhang Y Li J Chen H. Fluorescent reconstitution on deposition of PM_2.5_ in lung and extrapulmonary organs. Proc Natl Acad Sci USA. 2019;116:2488–2493. 30692265 10.1073/pnas.1818134116 PMC6377456

[32] Pan D-H Sheng J Wang X-Y. In vivo SPECT imaging of an ^131^I-labeled PM_2.5_ mimic substitute. Nucl Sci Tech. 2020;31:111.

[33] Lu K Zha Y Dong S. Uptake route altered the bioavailability of graphene in *Misgurnus anguillicaudatus*: comparing waterborne and sediment exposures. Environ Sci Technol. 2022;56:9435–9445. 35700278 10.1021/acs.est.2c01805

[34] Jasim DA Boutin H Fairclough M. Thickness of functionalized graphene oxide sheets plays critical role in tissue accumulation and urinary excretion: a pilot PET/CT study. Appl Mater Today. 2016;4:24–30.

[35] Jasim DA Ménard-Moyon C Bégin D Bianco A Kostarelos K. Tissue distribution and urinary excretion of intravenously administered chemically functionalized graphene oxide sheets. Chem Sci. 2015;6:3952–3964. 28717461 10.1039/c5sc00114e PMC5497267

[36] Jasim DA Newman L Rodrigues AF. The impact of graphene oxide sheet lateral dimensions on their pharmacokinetic and tissue distribution profiles in mice. J Control Release. 2021;338:330–340. 34418522 10.1016/j.jconrel.2021.08.028

[37] Li B Yang J Huang Q. Biodistribution and pulmonary toxicity of intratracheally instilled graphene oxide in mice. NPG Asia Mater. 2013;5:e44.

[38] Mao L Hu M Pan B Xie Y Petersen EJ. Biodistribution and toxicity of radiolabeled few layer graphene in mice after intratracheal instillation. Part Fibre Toxicol. 2016;13:7. 26864058 10.1186/s12989-016-0120-1 PMC4750184

[39] Cazier H Malgorn C Georgin D. Correlative radioimaging and mass spectrometry imaging: a powerful combination to study ^14^C-graphene oxide in vivo biodistribution. Nanoscale. 2023;15:5510–5518. 36853236 10.1039/d2nr06753f

[40] Al-Sid-Cheikh M Rouleau C Bussolaro D Oliveira Ribeiro CA Pelletier E. Tissue distribution of radiolabeled ^110m^Ag nanoparticles in fish: Arctic charr (*Salvelinus alpinus*). Environ Sci Technol. 2019;53:12043–12053. 31487449 10.1021/acs.est.9b04010

[41] Istiqola A Syafiuddin A. A review of silver nanoparticles in food packaging technologies: regulation, methods, properties, migration, and future challenges. J Chin Chem Soc (Taipei). 2020;67:1942–1956.

[42] Ke B Chen H Ma L. Visualization of mercury(II) accumulation in vivo using bioluminescence imaging with a highly selective probe. Org Biomol Chem. 2018;16:2388–2392. 29560483 10.1039/C8OB00398J

